# Child health and development in the course of the COVID-19 pandemic: are there social inequalities?

**DOI:** 10.1007/s00431-022-04799-9

**Published:** 2023-01-06

**Authors:** Simone Weyers, Mariann Rigó

**Affiliations:** Institute of Medical Sociology, Centre for Health and Society, Medical Faculty and University Hospital Düsseldorf, Heinrich-Heine-University, Düsseldorf, Germany

**Keywords:** Child health inequalities, Overweight, Motor development, Language development

## Abstract

COVID-19 pandemic lockdowns and closures have influenced all children’s health and development (HAD). We aimed to examine whether this differs by social circumstances. We hypothesised that socially disadvantaged children experienced more pronounced deterioration in their HAD compared with better-off children. In our trend study, we included five cohorts of school enrolment medical screening (school entrance 2018–2022) in the city of Dusseldorf, Germany. To compensate for selection bias due to the limited number of examinations in pandemic months, we chose the first 800 examinations of each cohort. We computed predicted prevalences of overweight, coordination and language problems as indicators of HAD. Neighbourhood deprivation, single-parent families and non-German nationality were used as indicators of social disadvantage. All groups of children experienced a deterioration in their HAD. Its magnitude is comparable between children with different social circumstances. For instance, between 2018 and 2021, prevalence of overweight increased from 19.2 to 24.2% in children from deprived neighbourhoods, and from 8.1 to 16.5% in children from well-off neighbourhoods. Prevalence of language problems (prepositions) increased from 49.9 to 72.1% among non-German children, and from 14.4 to 39.1% among German children.

*Conclusion*: Results only partly confirmed our hypothesis. However, since the pre-pandemic prevalences of HAD problems among disadvantaged children were already higher, further deterioration — regardless of its magnitude — has led to their particularly poor situation. For those children, overweight and language development should be priorities of prevention. Generally, important settings for child HAD such as kindergartens or leisure facilities should remain open in future pandemics.

**Table:** 

**What is known:** *• The COVID-19 pandemic has had a negative impact on general child health and development.* *• Before the pandemic, social inequalities in child health and development were a common phenomenon.*	
**What is new:** *• Children in all social groups experienced a deterioration in their health and development over the course of the pandemic.* *• Over the course of the pandemic, children from deprived neighbourhoods have demonstrated a particularly high prevalence of overweight. Language problems are particularly prevalent among non-German children.*

**Supplementary Information:**

The online version contains supplementary material available at 10.1007/s00431-022-04799-9.

## Introduction

The COVID-19 pandemic with its lockdowns and closures of kindergartens, schools and leisure facilities has influenced child health and development (HAD) in many ways — as has meanwhile been shown by individual studies and reviews: mental disorders have increased [1]; motor skills have been inhibited and overall physical fitness has worsened [2]; children have gained weight [3, 4]; language development has worsened [5, 6]. Social inequalities in child HAD are a common pattern [7, 8], and it has been postulated that children from vulnerable backgrounds will be most affected by the pandemic. [9, 10] However, studies empirically investigating the consequences of the COVID-19-pandemic on child HAD seldom apply an inequalities perspective. There are some exceptions e.g. survey data showed that vulnerable children were at increased risk of mental health impairments [11, 12] and of decreasing motor performance [13]; hospital patient data showed that social disparities in overweight and obesity have increased since the onset of the pandemic [14]; data of school enrolment examinations showed that children with low parental education and those with a migration background have a higher increase in overweight and language problems than their reference groups [5].

Given the research gap of a social-differential analysis of the impact of the pandemic on children’s HAD, we aim to examine the changes in children’s development by social circumstances throughout the pandemic. We hypothesise that socially disadvantaged children experienced more pronounced deterioration in their HAD compared to better-off children.

## Materials and methods

Our trend study is based on the school enrolment medical screening of children in the city of Dusseldorf, western Germany. This examination is mandatory for all children before school entry (both into public and private education) with approx. 6 years, and it is conducted by the municipal health authorities. The school enrolment medical screening is a valuable data source in the context of child health inequalities since it reaches children and their families with different social circumstances [15]. Child health and development is assessed by means of social-paediatric screening including different dimensions of school readiness [16]. Here, each child undergoes a range of standardised tests.

For the present trend study, we included five cohorts of pre-schoolers (wave 1 (w1): school year 2018/19 – wave 5 (w5): school year 2022/23). We received anonymised data sets from the health authorities.

For the analyses, we used three different indicators of child development (dependent variables) that had been measured by medical officers in a standardised way [17]: overweight was operationalised as gender-specific BMI > 90th percentile according to Kromeyer-Hauschild et al. [18]; body coordination was measured by lateral jumps; language development was measured by using correct preposition and plural formation when completing sentences. We dichotomised the latter three measures based on the number of points achieved by the child during the examinations. We used cut-off values indicated by the medical officer [6]: in the case of the coordination exercise, problematic development was indicated if the child achieved fewer than 7 points out of 32. For the two language exercises, 3 or less (plural) or 4 or less (prepositions) points achieved out of 7 (plural) or 8 (prepositions) indicated problematic development.

We also used three indicators of the child’s social circumstances (independent variables) that had been assessed by medical nurses: (i) neighbourhood deprivation served as a proxy of the family’s socio-economic position (SEP) [19]. It had been assigned via the child’s residential address and neighbourhood, based on indicators such as welfare benefits, living space per person and migration population on neighbourhood level. It ranges from 1 (no deprivation) to 5 (very high deprivation) and we compared 4/5 (high/very high deprivation) to 1–3 (no to medium deprivation); (ii) with regard to the family status we contrasted single-parent with two-parent families; (iii) for migration background, we compared children with non-German nationality to those with German nationality.

An important specificity of the examination procedure is that vulnerable children (with health problems or from disadvantaged neighbourhoods) are usually examined with priority in each cohort. Due to the limited number of examinations in the pandemic months, this resulted in smaller samples with a selection bias towards vulnerable children in the waves of 2020/2021 and 2021/2022. In order to analyse the development of children on comparable samples throughout our study period, we restricted our sample to the first 800 children of each cohort. A similar approach was chosen in an earlier work of Bredahl analysing the same data [6].

First, we calculated absolute and relative frequencies of independent and dependent variables for the first 800 children of each cohort. Then we analysed the trend in the development of child health by calculating the average predicted probabilities of detecting problematic health development. We estimated logistic regression models separately for each examination type (overweight, coordination, language), adjusted for sex, neighbourhood deprivation, family status and nationality. To examine whether time trends differ by children’s social status, we added an interaction term into the regressions composed of wave dummies and the indicator of social status. As the estimated regression coefficients are not straightforward to interpret, we present the predicted probabilities of detecting health problems by various indicators of social status. Furthermore, we computed the average marginal effect (AME) of a change between social circumstances. This indicates, for instance in the case of family status and overweight, the difference in predicted probabilities of detecting overweight between children in single-parent and two-parent families. Additionally, we computed the AME of a wave change within the social groups showing the difference in predicted prevalences over time. When interpreting the results, we placed special focus on the AME between w4 and w1 as we expect that the school cohort 21/22 (those starting school in September 2021) was most adversely affected by the lockdown and the following pandemic measures. The pre-schoolers of cohort 21/22 were in the middle of their kindergarten years when the lockdown started, so they spent the most intensive pre-school months being faced with pandemic measures. On the other hand, the following cohort of 22/23 enjoyed a relatively pandemic-free pre-school year. All analyses were conducted using Stata 16.

## Results

Table 1 depicts the descriptives of the data set used for the analyses. The unadjusted prevalence of overweight in the prepandemic w1 is 12.5%. Coordination problems are detected among 6.4% of the children, while 17.5% display language problems in terms of preposition formation and 18.9% in terms of plural formation. Substantially higher prevalences are observed in the pre-schoolers of w4 with 21.4% overweight, 20.1% with coordination problems, 48.9% with language problems/preposition and 45.8% with language problems/plural formation.

**Table 1:** Descriptive statistics of the study populations of five cohorts of preschoolers used in the analyses

	**Wave 1**		**Wave 2**		**Wave 3**		**Wave 4**		**Wave 5**		**Total**	
**school year**	**18/19**		**19/20**		**20/21**		**21/22**		**22/23**			
	*No.*	*%*	*No.*	*%*	*No.*	*%*	*No.*	*%*	*No.*	*%*	*No.*	*%*
**Overweight**												
no	659	87.5	669	85.3	660	83.8	614	78.6	661	83.9	3,263	83.8
yes	94	12.5	115	14.7	128	16.2	167	21.4	127	16.1	631	16.2
Total	753	100	784	100	788	100	781	100	788	100	3,894	100
**Coordination problems**												
no	686	93.6	654	87.6	645	89.2	494	79.9	550	85.4	3,029	87.4
yes	47	6.4	93	12.4	78	10.8	124	20.1	94	14.6	436	12.6
Total	733	100	747	100	723	100	618	100	644	100	3,465	100
**Language problems (prepositions)**												
no	590	82.5	487	67	480	67.2	336	51.1	352	52.5	2,245	64.4
yes	125	17.5	240	33	234	32.8	322	48.9	319	47.5	1,240	35.6
Total	715	100	727	100	714	100	658	100	671	100	3,485	100
**Language problems (plural)**												
no	575	81.1	479	66.9	488	69.8	351	54.2	381	57.7	2,274	66.3
yes	134	18.9	237	33.1	211	30.2	297	45.8	279	42.3	1,158	33.7
Total	709	100	716	100	699	100	648	100	660	100	3,432	100
**Neighbourhood**												
well-off	481	60.2	309	38.9	357	45.2	347	44.4	374	47.0	1,868	47.2
deprived	318	39.8	485	61.1	432	54.8	435	55.6	421	53.0	2,091	52.8
Total	799	100	794	100	789	100	782	100	795	100	3,959	100
**Single-parent family**												
no	684	85.5	663	83.0	672	84.0	661	82.6	666	83.3	3,346	83.7
yes	116	14.5	136	17.0	128	16.0	139	17.4	134	16.8	653	16.3
Total	800	100	799	100	800	100	800	100	800	100	3,999	100
**Nationality**												
German	667	83.4	525	66.0	597	75.0	555	69.5	506	67.6	2,850	72.4
other	133	16.6	271	34.0	199	25.0	243	30.5	242	32.4	1,088	27.6
Total	800	100	796	100	796	100	798	100	748	100	3,938	100

Based on our logistic regression estimations stratifying by indicators of social disadvantage, we observe the following results (see all results in table 2, electronic supplement).

### Neighbourhood

**Fig. 1:**
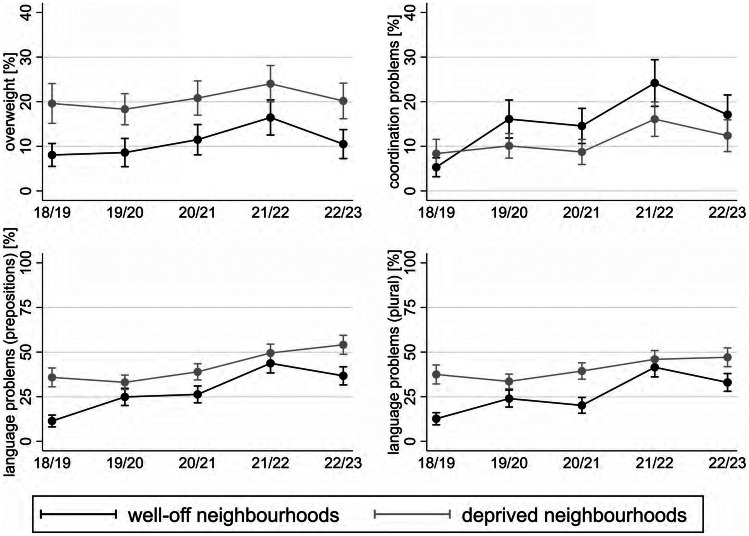
Predicted prevalences of overweight, coordination and language problems by neighbourhood deprivation and school year

Results indicate a deteriorating trend of overweight in both groups of children (Fig. 1). The magnitude of the change over time (difference w4–w1) is greater in children from well-off neighbourhoods (AME = 8.4; *p* = .000) compared to those from deprived neighbourhoods (AME = 5.0; *p* = .102). The corresponding AMEs of a wave change between w4 and w1 are indicated by the last column of supplementary table 2. For instance, the AME of 5.0 in case of deprived neighbourhoods shows the difference between the predicted prevalence of overweight in w4 (24.2) and w1 (19.2). However, our calculations imply a higher prevalence of overweight among children from deprived neighbourhoods with predicted probability of 24.2% in w4 compared to 16.5% in children from well-off neighbourhoods (AME = 7.7; *p* = .008). Regarding coordination problems, a significant increase is observed in both groups of children from w1 to w4 (AME = 19.0 in children from well-off neighbourhoods; AME = 7.7 in children from deprived neighbourhoods). Also, contrary to our hypothesis, children in well-off neighbourhoods have a higher prevalence in most waves. Regarding language problems, we detect worsening trends in both groups of children. While the magnitude of deterioration is larger among children from well-off neighbourhoods (AME = 32.5; *p* = .000 in prepositions; AME = 29.0 in plural; *p* = .000), the probability of developing language problems also significantly increases among children in deprived neighbourhoods (AME = 14.9; *p* = 0.000 in prepositions; AME = 9.4; p = 0.011 in plural). The significant gaps in prior waves disappear in w4 (AME = 6.0; *p* = .108 in prepositions; AME = 4.7; *p* = .206 in plural). In w5, children from well-off neighbourhoods relief, while no improvement is observed among disadvantaged children. They experience a worsening trend at a higher level.

### Family status

**Fig. 2:**
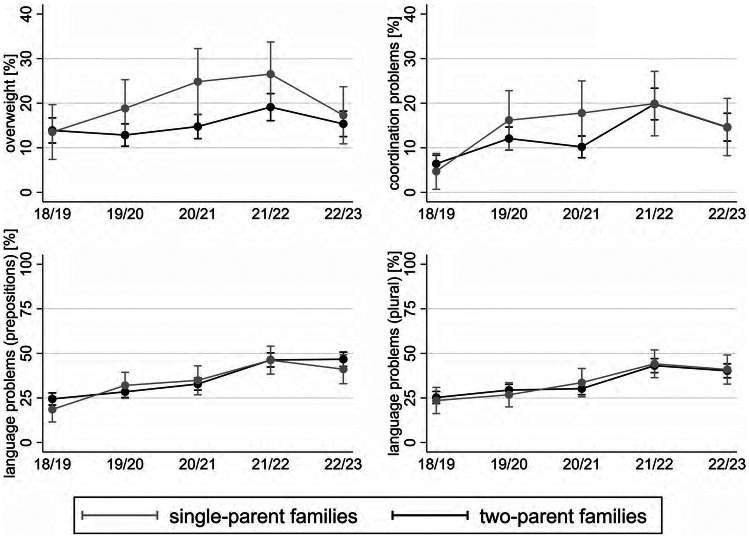
Predicted prevalences of overweight, coordination and language problems by family status and school year

Results indicate a parallel deteriorating trend and no significant differences between single- and two-parent families in the case of coordination and language problems (Fig. 2). However, regarding overweight, results point to a significant worsening trend among children in single-parent families (increasing from 13.4% in w1 to 26.6% in w4), which is greater in magnitude (AME = 13.1) than the one detected among children in two-parent families (AME = 5.5). After reaching the peak in w4, an improvement took place in both groups.

### Nationality

**Fig. 3:**
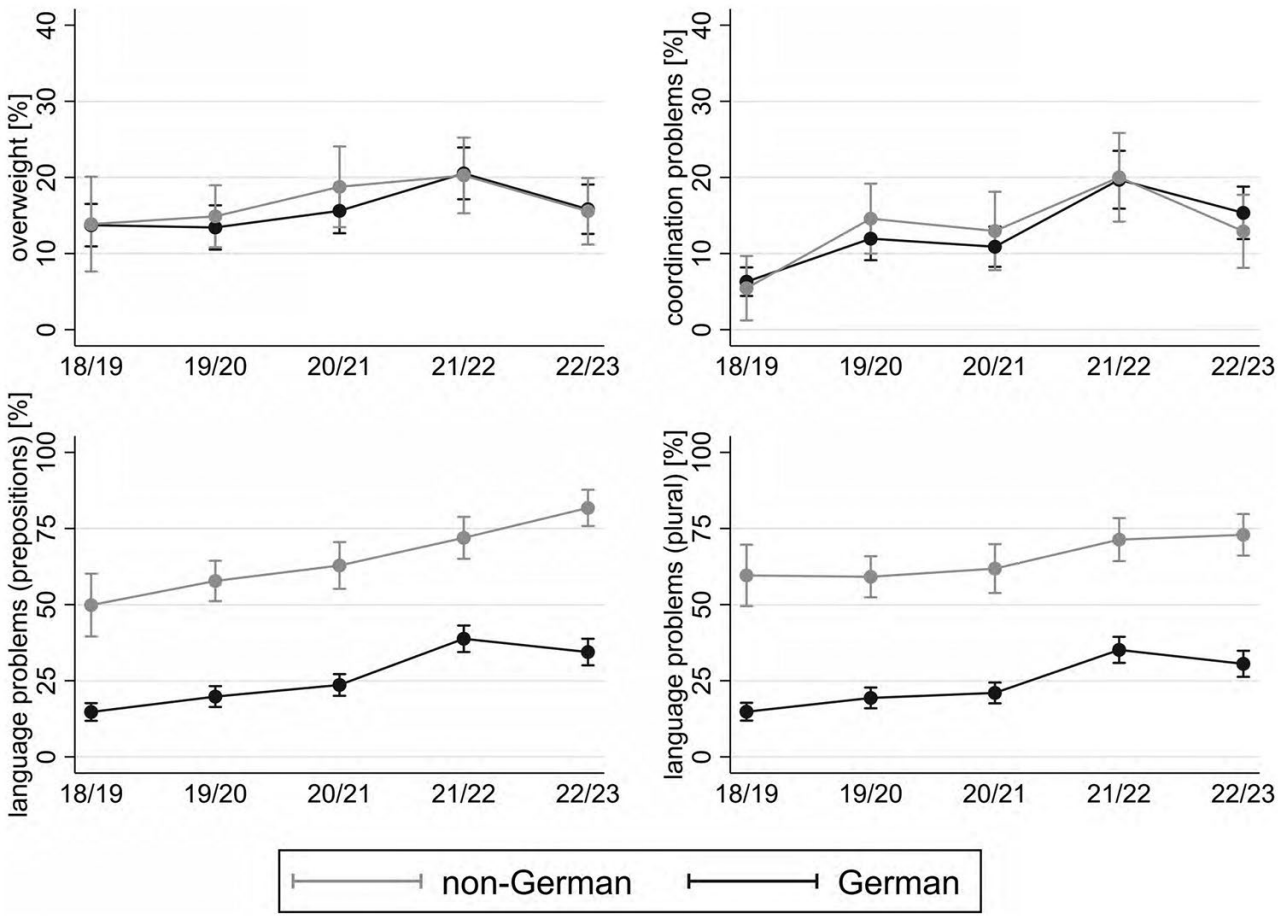
Predicted prevalences of overweight, coordination and language problems by nationality and school year

Focusing on overweight and coordination problems, results indicate a worsening trend in both groups (Fig. 3). However, no significant differences are found between German vs. non-German children. Regarding language problems, the results point to a marked disadvantage of children in non-German families in each wave. The corresponding AMEs displaying the difference in the predicted prevalences of language problems in each wave lie between the interval of 33.0 and 47.3. Over time, both groups experience an increase in the prevalence of language problems. However, in case of prepositions, it reaches its peak at 81.7% in w5 in non-German families, while peaking at 39.1% in w4 followed by a subsequent improvement in w5 in German families.

## Discussion

The aim of the study was to examine the changes in children’s development by social circumstances throughout the pandemic. We hypothesised that socially disadvantaged children experienced more pronounced deterioration in their development compared to those with better social circumstances.

Our results only partly support our hypothesis. All groups of children experienced a deterioration in their development over the course of the pandemic. The magnitude of deterioration is in most cases comparable between social groups, or, in some cases, even smaller among children in worse social circumstances. However, bearing in mind that the prevalence of developmental problems among disadvantaged children is already higher w1, further deterioration — regardless of its magnitude — has led to their particularly poor situation. Similar observation was made in a previous study of our group regarding overweight, parental education and neighbourhood deprivation [19].

### Overweight

Our results show marked inequalities of overweight even before the pandemic by neighbourhood deprivation as a proxy of the family’s socio-economic position. This is in line with previous well-established findings indicating that both a family’s SEP and neighbourhood environment are associated with child overweight [20–22]. Our results regarding how the pandemic impacted children’s HAD are in line with the study of Jenssen et al. [14] showing that the increase in overweight was more pronounced in children with lower family income; and correspond to the study of Bantel [5] documenting a greater increase in children with low parental education. However, we did not observe inequalities according to migration background that Jenssen et al. [14] found with regard to Hispanic and Black patients vs. White patients. Differences in physical activity and nutrition are key factors to explain these socio-economic inequalities in child overweight during the pandemic. During closures of kindergartens, all children suffered from a lack of active movement and exercise. However, socio-economically disadvantaged children additionally lacked compensating facilities such as gardens [23] and green spaces [24] at home. Studies show that apartments and proximity to major roads correlated with children’s decreased outdoor activities during the pandemic [25]. Furthermore, kindergartens provide access to healthy food for many children growing up in families with income insecurity. During lockdown these children lost access to setting-based nutrition programmes [3] and they were restricted to their home environments with increased food intake and unhealthy food choices [4]. It is also an interesting finding that the prevalence of overweight decreases for all children from wave 4 to wave 5. This might indicate the important role of settings such as kindergartens and leisure facilities for physical activity and nutrition.

### Coordination problems

Our findings do not respond to the study of Wessely et al. [13] who found a steeper decrease in lateral jumping performance in children with higher social burden. Interestingly, our results point to the opposite direction with smaller increase of coordination problems among socially disadvantaged children.

### Language

Our results correspond to the study of Bantel [5] where the increase in language difficulties was mainly observed in pre-schoolers with non-German origin. This effect can mainly be attributed to the closure of kindergartens. For children with a migration background, the kindergarten provides access to the host country’s language and education system, and it prevents language deficits. This has been previously shown by school enrolment examinations documenting that deficits in German language skills are less likely among migrant children the longer they have attended the kindergarten [26]. Thus, for these children language development during the pandemic was dependent on social interaction with family members who are likely to have a foreign mother tongue as well. Media consumption aggravated this problem. Children’s screen time has increased globally during the pandemic [27, 28]. But the massive screen time was probably different in children growing up with a foreign mother tongue compared with native German speakers. Ritterfeld et al. [29] have shown that children growing up multilingually use less language-driven, but more image-driven media.

## Strengths and limitations

An important strength of the study is that it is based on actual administrative data involving children from all social groups. This provides a unique opportunity to analyse inequalities of child HAD in a timely and efficient manner. Within these parameters, four indicators of child HAD could be observed that had been measured by standard medical procedure. However, as socio-emotional development had not been assessed in the present school enrolment medical screening, we could not include this variable of child development in our analyses. Another strength is that three different indicators of social circumstances could be included in the analyses, making it possible to show their different relationships with health. These are, however, methodologically limited: (i) neighbourhood deprivation is only a proxy of the family’s socio-economic position with the risk of ecological fallacy. Due to data protection, no individual level SEP-data were available; (ii) regarding migration background, information about parents’ nationality should also be included for proper measurement [30], as well as information on the family’s language [31]. However, none of this information was available in the administrative data. Similarly, the number of available confounders is also limited: beside the few variables describing the children’s social circumstances, the school enrolment medical examinations do not assess detailed information on their personal and family circumstances (e.g. living environment, occupational situation) and health behaviour (e.g. physical activity). Therefore, we could not adjust our regressions for these variables.

A further limitation might relate to possible gender differences in trends by social groups. However, we have checked if the general trends of the outcome variables differ by sex. The interactions terms between the wave and sex dummies were not significant in either case. Furthermore, differences in predicted prevalences by sex were in most cases statistically not significant. Therefore, to enhance the precision of the estimates, we decided to include sex only as a control variable and assumed that trends within social groups are not different by gender.

A central limitation of the analysis relates to our sample: due to the pandemic, there was a marked drop in the number of examinations and they were mainly focused on vulnerable children. In the pre-pandemic w1 and w2 4891 and 4564 children were examined, while in the pandemic w3 and w4 the number of examinations was 2767 and 1754 respectively. This also seriously affected the composition of the pandemic w3 and w4, which mainly included the vulnerable children. The composition of the total surveyed population in each wave is described by supplementary table 3. It should be noted that examinations take place in a prioritised order each year, with vulnerable children being invited first to attend. Therefore, to alleviate the sample selection bias, we restricted our sample to the first 800 examinations in each wave corresponding to Bredahl [6]. We chose a different approach than Bantel et al. [5] since we believe that cutting the sample size by the date of examination instead of weighting leads to a more homogeneous sample. However, an important consequence of this procedure is that our results apply mainly to the group of vulnerable children and cannot be generalised to the total sample of pre-school children. Most children with a favourable family background and without medical indication are not observed in the data. These children were shown to be less disadvantaged during the pandemic in previous literature as mentioned above. Therefore, we expect that the gap between children with different social circumstances is larger in the total sample than the one we observed in our dataset.

## Conclusions

The massive excess of post-pandemic language problems in children with non-German mother tongue in our study population suggests what happens if the kindergarten is closed for a longer period. Since language acquisition is a key task in pre-school age and it is essential for cognitive and psychosocial development, we conclude that kindergartens should remain available for all children in future epidemic scenarios.

The coexistence of the COVID-19 pandemic and the overweight pandemic has been mentioned by several authors [26, 32]. Given the long-term consequences of child overweight for adult health [33] and the social inequalities observed here, post-pandemic resources should be invested in prevention programmes for vulnerable children. Setting-based approaches in kindergartens [34] and schools [35] are important vehicles. Small-scale analysis of social structures can identify particularly deprived urban neighbourhoods to focus on [19]. In addition, policies to reduce social inequalities in health should not be forgotten, in line with the framework of social determinants of health and inequalities [36].

## Supplementary Information

Below is the link to the electronic supplementary material.

Supplementary file1 (DOCX 36 KB)

Supplementary file2 (DOCX 26 KB)

## Abbreviations

AME: Average marginal effect
HAD: Health and development
SEP: Socio-economic position
w: Wave
