# Supplementary material for: Child health and development in the course of the COVID-19 pandemic: are there social inequalities?
Source: Eur J Pediatr. 2023 Jan 6;182(3):1173–81. doi: 10.1007/s00431-022-04799-9 (PMC9816013; doi:10.1007/s00431-022-04799-9)
Supplement: Supplementary file 1 — Supplementary file1 (DOCX 36 KB) [file 431_2022_4799_MOESM1_ESM.docx]

Supplementary Table 2a: predicted prevalences of HAD problems (%) by neighbourhood and AMEs

| **Neighbourhood** | | | | | | |
| --- | --- | --- | --- | --- | --- | --- |
|  | **Wave 1** | **Wave 2** | **Wave 3** | **Wave 4** | **Wave 5** | **AME**  **W4 vs. W1**  **(p-value)** |
| **school year** | **18/19** | **19/20** | **20/21** | **21/22** | **22/23** |  |
| **Overweight (N=3,801)** | | | | | | |
| **well-off** | 8.1 | 8.7 | 11.4 | 16.5 | 10.5 | 8.4 |
|  | (5.5 - 10.7) | (5.5 - 11.9) | (8.0 - 14.8) | (1.26 - 20.5) | (7.2 - 13.7) | (0.000) |
| **deprived** | 19.2 | 18.2 | 20.9 | 24.2 | 19.8 | 5.0 |
|  | (14.8 - 23.6) | (14.7 - 21.7) | (17.0 - 24.7) | (20.1 - 28.3) | (15.8 - 23.7) | (0.102) |
| **AME deprived vs. well-off (p-value)** | 11.1 | 9.5 | 9.4 | 7.7 | 9.3 |  |
|  | (0.000) | (0.000) | (0.000) | (0.008) | (0.000) |  |
| **Coordination problem (N=3,390)** | | | | | | |
| **well-off** | 5.3 | 16.3 | 14.5 | 24.3 | 17.1 | 19.0 |
|  | (3.2 - 7.5) | (12.0 - 20.6) | (10.6 - 18.4) | (19.0 - 29.6) | (12.7 - 21.5) | (0.000) |
| **deprived** | 8.3 | 10.0 | 8.5 | 16.0 | 12.5 | 7.7 |
|  | (5.1 - 11.5) | (7.3 - 12.8) | (5.7 - 11.3) | (12.2 - 19.9) | (8.9 - 16.1) | (0.002) |
| **AME deprived vs. well-off (p-value)** | 3.0 | -6.2 | -6.0 | -8.3 | -4.6 |  |
|  | (0.127) | (0.017) | (0.015) | (0.013) | (0.113) |  |
| **Language problem (prepositions) (N=3,401)** | | | | | | |
| **well-off** | 11.4 | 24.8 | 26.2 | 43.9 | 36.9 | 32.5 |
|  | (8.1 - 14.7) | (19.9 - 29.6) | (21.5 - 30.9) | (38.5 - 49.3) | (31.8 - 42.0) | (0.000) |
| **deprived** | 35.0 | 32.8 | 39.1 | 49.9 | 53.9 | 14.9 |
|  | (29.8 - 40.2) | (28.8 - 36.8) | (34.5 - 43.6) | (45.0 - 54.9) | (48.6 - 59.2) | (0.000) |
| **AME deprived vs. well-off (p-value)** | 23.6 | 8.0 | 12.9 | 6.0 | 17.0 |  |
|  | (0.000) | (0.013) | (0.000) | (0.108) | (0.000) |  |
| **Language problem (plural) (N=3,349)** | | | | | | |
| **well-off** | 12.7 | 23.8 | 20.1 | 41.7 | 33.2 | 29.0 |
|  | (9.3 - 16.1) | (19.1 - 28.6) | (15.7 - 24.5) | (36.3 - 47.1) | (28.2 - 38.2) | (0.000) |
| **deprived** | 37.0 | 33.0 | 39.5 | 46.4 | 46.9 | 9.4 |
|  | (31.7 - 42.3) | (29.0 - 37.1) | (34.9 - 44.1) | (41.5 - 51.3) | (41.6 - 52.2) | (0.011) |
| **AME deprived vs. well-off (p-value)** | 24.3 | 9.2 | 19.4 | 4.7 | 13.7 |  |
|  | (0.000) | (0.004) | (0.000) | (0.206) | (0.000) |  |

*Notes: Predicted prevalences based on logistic regressions. Values expressed in %. Right-hand side variables included in the logistic regression: two-way interactions of neighbourhood deprivation and wave dummies, nationality, family status, gender. 95% confidence intervals in parenthesis. AME (average marginal effect): p-values in parenthesis. Sample: first 800 examinations in each wave.*

Supplementary Table 2b: predicted prevalences of HAD problems (%) by family status and AMEs

| **Family status** | | | | | | |
| --- | --- | --- | --- | --- | --- | --- |
|  | **Wave 1** | **Wave 2** | **Wave 3** | **Wave 4** | **Wave 5** | **AME**  **W4 vs. W1**  **(p-value)** |
| **school year** | **18/19** | **19/20** | **20/21** | **21/22** | **22/23** |  |
| **Overweight (N=3,801)** | | | | | | |
| **two-parent family** | 13.7 | 12.9 | 14.8 | 19.3 | 15.2 | 5.5 |
|  | (10.9 - 16.6) | (10.4 - 15.4) | (12.1 - 17.5) | (16.2 - 22.3) | (12.4 - 18.0) | (0.009) |
| **single-parent family** | 13.4 | 18.5 | 24.9 | 26.6 | 16.7 | 13.1 |
|  | (7.3 - 19.5) | (12.2 - 24.9) | (17.4 - 32.3) | (19.4 - 33.8) | (10.4 - 23.1) | (0.006) |
| **AME single vs. two**  **(p-value)** | -0.3 | 5.6 | 10.1 | 7.3 | 1.5 |  |
|  | (0.923) | (0.106) | (0.012) | (0.068) | (0.666) |  |
| **Coordination problem (N=3,390)** | | | | | | |
| **two-parent family** | 6.4 | 12.1 | 10.0 | 19.8 | 14.6 | 13.4 |
|  | (4.5 - 8.3) | (9.5 - 14.7) | (7.6 - 12.4) | (16.3 - 23.4) | (11.5 - 17.7) | (0.000) |
| **single-parent family** | 4.7 | 16.1 | 17.8 | 19.9 | 14.8 | 15.3 |
|  | (0.7 - 8.7) | (9.5 - 22.7) | (10.6 - 25.0) | (12.7 - 27.2) | (8.3 - 21.3) | (0.000) |
| **AME single vs. two**  **(p-value)** | -1.8 | 4.0 | 7.8 | 0.1 | 0.2 |  |
|  | (0.434) | (0.268) | (0.044) | (0.983) | (0.958) |  |
| **Language problem (prepositions) (N=3,401)** | | | | | | |
| **two-parent family** | 24.1 | 28.1 | 32.9 | 46.7 | 46.7 | 22.6 |
|  | (20.7 - 27.5) | (24.9 - 31.3) | (29.4 - 36.3) | (42.7 - 50.7) | (42.7 - 50.7) | (0.000) |
| **single-parent family** | 18.5 | 32.6 | 34.9 | 46.3 | 41.0 | 27.8 |
|  | (11.4 - 25.5) | (25.3 - 39.9) | (26.8 - 43.1) | (38.4 - 54.1) | (32.9 - 49.2) | (0.000) |
| **AME single vs. two**  **(p-value)** | -5.6 | 4.6 | 2.1 | -0.4 | -5.7 |  |
|  | (0.157) | (0.262) | (0.641) | (0.924) | (0.219) |  |
| **Language problem (plural) (N=3,349)** | | | | | | |
| **two-parent family** | 25.1 | 29.0 | 30.3 | 43.5 | 40.3 | 18.4 |
|  | (21.7 - 28.5) | (25.8 - 32.2) | (26.9 - 33.7) | (39.6 - 47.4) | (36.4 - 44.2) | (0.000) |
| **single-parent family** | 23.4 | 26.7 | 33.7 | 44.2 | 40.9 | 20.8 |
|  | (16.1 - 30.7) | (19.9 - 33.4) | (25.7 - 41.6) | (36.4 - 52.0) | (32.7 - 49.0) | (0.000) |
| **AME single vs. two**  **(p-value)** | -1.7 | -2.4 | 3.4 | 0.7 | 0.6 |  |
|  | (0.679) | (0.534) | (0.442) | (0.871) | (0.905) |  |

*Notes: Predicted prevalences based on logistic regressions. Values expressed in %. Right-hand side variables included in the logistic regression: two-way interactions of family status and wave dummies, nationality, neighbourhood deprivation, gender. 95% confidence intervals in parenthesis. AME (average marginal effect): p-values in parenthesis. Sample: first 800 examinations in each wave.*

Supplementary Table 2c: predicted prevalences of HAD problems (%) by nationality and AMEs

| **Nationality** | | | | | | |
| --- | --- | --- | --- | --- | --- | --- |
|  | **Wave 1** | **Wave 2** | **Wave 3** | **Wave 4** | **Wave 5** | **AME**  **W4 vs. W1**  **(p-value)** |
| **school year** | **18/19** | **19/20** | **20/21** | **21/22** | **22/23** |  |
| **Overweight (N=3,801)** | | | | | | |
| **German** | 13.5 | 13.5 | 15.6 | 20.6 | 15.6 | 7.0 |
|  | (10.8 - 16.3) | (10.6 - 16.4) | (12.7 - 18.6) | (17.2 - 24.0) | (12.4 - 18.8) | (0.002) |
| **non-German** | 13.9 | 14.8 | 18.8 | 20.6 | 15.3 | 6.7 |
|  | (7.6 - 20.1) | (10.7 - 18.8) | (13.5 - 24.1) | (15.6 - 25.6) | (11.0 - 19.7) | (0.098) |
| **AME non-German vs. German**  **(p-value)** | 0.3 | 1.3 | 3.2 | 0.0 | -0.3 |  |
|  | (0.924) | (0.620) | (0.305) | (0.994) | (0.922) |  |
| **Coordination problem (N=3,390)** | | | | | | |
| **German** | 6.3 | 12.0 | 10.7 | 19.8 | 15.3 | 13.5 |
|  | (4.4 - 8.2) | (9.2 - 14.9) | (8.1 - 13.3) | (16.0 - 23.6) | (11.9 - 18.7) | (0.000) |
| **non-German** | 5.4 | 14.5 | 13.0 | 20.0 | 13.1 | 14.5 |
|  | (1.2 - 9.7) | (9.9 - 19.1) | (7.8 - 18.1) | (14.1 - 25.8) | (8.2 - 18.0) | (0.000) |
| **AME non-German vs. German**  **(p-value)** | -0.9 | 2.5 | 2.3 | 0.2 | -2.2 |  |
|  | (0.718) | (0.366) | (0.432) | (0.959) | (0.474) |  |
| **Language problem (prepositions) (N=3,401)** | | | | | | |
| **German** | 14.4 | 19.7 | 23.6 | 39.1 | 34.4 | 24.7 |
|  | (11.5 - 17.2) | (16.3 - 23.1) | (20.0 - 27.2) | (34.7 - 43.5) | (30.0 - 38.8) | (0.000) |
| **non-German** | 49.9 | 57.1 | 62.9 | 72.1 | 81.7 | 22.2 |
|  | (39.6 - 60.2) | (50.5 - 63.7) | (55.3 - 70.6) | (65.3 - 79.0) | (75.8 - 87.7) | (0.000) |
| **AME non-German vs. German**  **(p-value)** | 35.5 | 37.4 | 39.3 | 33.0 | 47.3 |  |
|  | (0.000) | (0.000) | (0.000) | (0.000) | (0.000) |  |
| **Language problem (plural) (N=3,349)** | | | | | | |
| **German** | 14.6 | 19.1 | 21.0 | 35.4 | 30.5 | 20.8 |
|  | (11.7 - 17.5) | (15.7 - 22.5) | (17.6 - 24.4) | (31.1 - 39.7) | (26.3 - 34.8) | (0.000) |
| **non-German** | 59.6 | 58.4 | 61.9 | 71.6 | 72.9 | 11.9 |
|  | (49.5 - 69.7) | (51.7 - 65.1) | (53.9 - 69.9) | (64.5 - 78.6) | (66.0 - 79.7) | (0.057) |
| **AME non-German vs. German**  **(p-value)** | 45.0 | 39.3 | 40.9 | 36.1 | 42.3 |  |
|  | (0.000) | (0.000) | (0.000) | (0.000) | (0.000) |  |

*Notes: Predicted prevalences based on logistic regressions. Values expressed in %. Right-hand side variables included in the logistic regression: two-way interactions of nationality and wave dummies, family status, neighbourhood deprivation, gender. 95% confidence intervals in parenthesis. AME (average marginal effect): p-values in parenthesis. Sample: first 800 examinations in each wave.*
