# Supplementary material for: Child health and development in the course of the COVID-19 pandemic: are there social inequalities?
Source: Eur J Pediatr. 2023 Jan 6;182(3):1173–81. doi: 10.1007/s00431-022-04799-9 (PMC9816013; doi:10.1007/s00431-022-04799-9)
Supplement: Supplementary file 2 — Supplementary file2 (DOCX 26 KB) [file 431_2022_4799_MOESM2_ESM.docx]

Supplementary Table 3: Descriptive statistics of the total populations of five cohorts of pre-schoolers

|  | **Wave 1** | | **Wave 2** | | **Wave 3** | | **Wave 4** | | **Wave 5** | | **Total** | |
| --- | --- | --- | --- | --- | --- | --- | --- | --- | --- | --- | --- | --- |
| **school year** | **18/19** | | **19/20** | | **20/21** | | **21/22** | | **22/23** | |  | |
|  | *No.* | *%* | *No.* | *%* | *No.* | *%* | *No.* | *%* | *No.* | *%* | *No.* | *%* |
| **Overweight** |  |  |  |  |  |  |  |  |  |  |  |  |
| no | 4,051 | 88.1 | 3,984 | 88.3 | 2,366 | 86.5 | 1,364 | 78.9 | 1,631 | 84.5 | 13,396 | 86.4 |
| yes | 545 | 11.9 | 528 | 11.7 | 368 | 13.5 | 364 | 21.1 | 299 | 15.5 | 2,104 | 13.6 |
| Total | 4,596 | 100 | 4,512 | 100 | 2,734 | 100 | 1,728 | 100 | 1,930 | 100 | 15,500 | 100 |
| **Coordination problems** | |  |  |  |  |  |  |  |  |  |  |  |
| no | 4,216 | 93.8 | 4,130 | 93.4 | 2,342 | 90.6 | 1,323 | 89.6 | 1,553 | 90.3 | 13,564 | 92.3 |
| yes | 279 | 6.2 | 294 | 6.6 | 242 | 9.4 | 153 | 10.4 | 166 | 9.7 | 1,134 | 7.7 |
| Total | 4,495 | 100 | 4,424 | 100 | 2,584 | 100 | 1,476 | 100 | 1,719 | 100 | 14,698 | 100 |
| **Language problems (prepositions)** | | |  |  |  |  |  |  |  |  |  |  |
| no | 3,605 | 81.7 | 3,412 | 79.5 | 1,816 | 70.5 | 957 | 61.7 | 1,110 | 62.7 | 10,900 | 74.7 |
| yes | 806 | 18.3 | 878 | 20.5 | 760 | 29.5 | 594 | 38.3 | 660 | 37.3 | 3,698 | 25.3 |
| Total | 4,411 | 100 | 4,290 | 100 | 2,576 | 100 | 1,551 | 100 | 1,770 | 100 | 14,598 | 100 |
| **Language problems (plural)** | |  |  |  |  |  |  |  |  |  |  |  |
| no | 3,590 | 81.9 | 3,340 | 79 | 1,841 | 72.6 | 983 | 64.4 | 1,144 | 65.4 | 10,898 | 75.6 |
| yes | 792 | 18.1 | 886 | 21 | 695 | 27.4 | 543 | 35.6 | 605 | 34.6 | 3,521 | 24.4 |
| Total | 4,382 | 100 | 4,226 | 100 | 2,536 | 100 | 1,526 | 100 | 1,749 | 100 | 14,419 | 100 |
| **Neighbourhood** |  |  |  |  |  |  |  |  |  |  |  |  |
| well-off | 3,444 | 70.9 | 2,980 | 66.0 | 1,233 | 45.3 | 584 | 33.8 | 830 | 42.6 | 9,071 | 57.5 |
| deprived | 1,413 | 29.1 | 1,538 | 34.0 | 1,490 | 54.7 | 1,144 | 66.2 | 1,120 | 57.4 | 6,705 | 42.5 |
| Total | 4,857 | 100 | 4,518 | 100 | 2,723 | 100 | 1,728 | 100 | 1,950 | 100 | 15,776 | 100 |
| **Single-parent family** |  |  |  |  |  |  |  |  |  |  |  |  |
| no | 4,153 | 84.9 | 3,843 | 84.2 | 2,338 | 84.5 | 1,428 | 81.4 | 1,673 | 84.8 | 13,435 | 84.2 |
| yes | 738 | 15.1 | 721 | 15.8 | 429 | 15.5 | 326 | 18.6 | 300 | 15.2 | 2,514 | 15.8 |
| Total | 4,891 | 100 | 4,564 | 100 | 2,767 | 100 | 1,754 | 100 | 1,973 | 100 | 15,949 | 100 |
| **Nationality** |  |  |  |  |  |  |  |  |  |  |  |  |
| German | 4,002 | 82.2 | 3,057 | 67.4 | 2,087 | 76.4 | 1,250 | 71.6 | 1,320 | 72.1 | 11,716 | 74.6 |
| other | 865 | 17.8 | 1,476 | 32.6 | 643 | 23.6 | 495 | 28.4 | 511 | 27.9 | 3,990 | 25.4 |
| Total | 4,867 | 100 | 4,533 | 100 | 2,730 | 100 | 1,745 | 100 | 1,831 | 100 | 15,706 | 100 |

*Notes: The dataset in wave 5 includes examinations only until April 2022.*
